# (1*S*,3*R*,8*R*,9*S*,11*R*)-2,2-Di­bromo-10,10-di­chloro-3,7,7,11-tetra­methyl­tetra­cyclo­[6.5.0.0^1,3^.0^9,11^]trideca­ne

**DOI:** 10.1107/S1600536813009070

**Published:** 2013-04-13

**Authors:** Najia Ourhriss, Ahmed Benharref, Mohamed Saadi, Moha Berraho, Lahcen El Ammari

**Affiliations:** a Laboratoire de Chimie Biomoléculaire, Substances Naturelles et Réactivité "Unité Associée au CNRST (URAC16)", Université Cadi Ayyad, Faculté des Sciences Semlalia, BP 2390, Bd My Abdellah, 40000 Marrakech, Morocco; b Laboratoire de Chimie du Solide Appliquée, Faculté des Sciences, Université Mohammed V-Agdal, Avenue Ibn Battouta, BP 1014, Rabat, Morocco

## Abstract

The title compound, C_17_H_24_Br_2_Cl_2_, was synthesized from β-himachalene (3,5,5,9-tetra­methyl-2,4a,5,6,7,8-hexa­hydro-1*H*-benzo­cyclo­heptene), which was isolated from the essential oil of the Atlas cedar (*Cedrus Atlantica*). The mol­ecule contains fused six-, seven- and two three-membered rings. The six-membered ring has a half-chair conformation, while the seven-membered ring displays a boat conformation. The absolute structure was unambiguously established from anomalous dispersion effects. The crystal packing exhibits no short inter­molecular contacts.

## Related literature

For the crystal structures of related compounds, see: Ourhriss *et al.* (2013 ▶); Oukhrib *et al.* (2013*a* ▶,*b* ▶). For puckering param­eters, see: Cremer & Pople (1975 ▶).
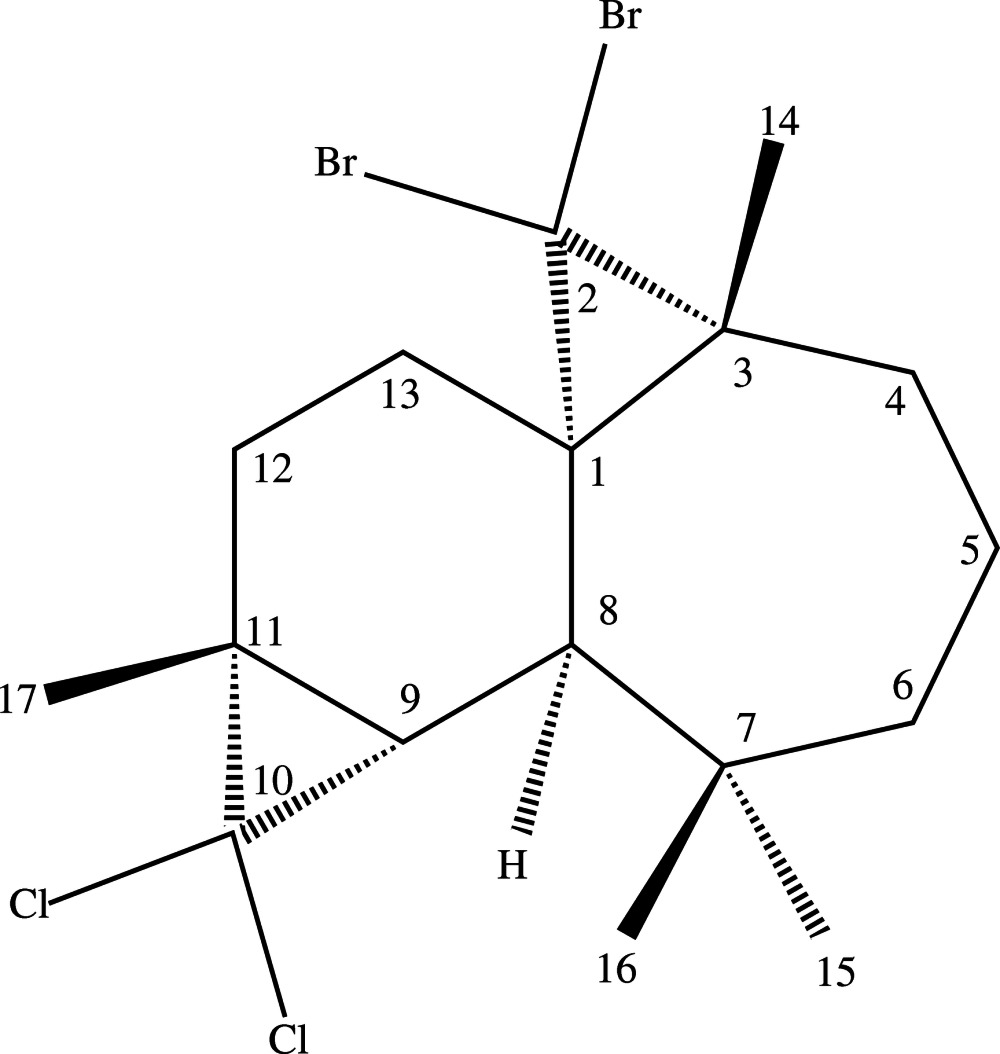


## Experimental

### 

#### Crystal data

C_17_H_24_Br_2_Cl_2_
*M* *_r_* = 459.08
Monoclinic, 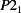

*a* = 9.0112 (6) Å
*b* = 11.6772 (8) Å
*c* = 9.0849 (6) Å
β = 108.045 (5)°
*V* = 908.94 (11) Å^3^
*Z* = 2
Mo *K*α radiation
μ = 4.75 mm^−1^
*T* = 296 K
0.45 × 0.34 × 0.29 mm

#### Data collection

Bruker X8 APEX diffractometer
Absorption correction: multi-scan (*SADABS*; Sheldrick, 2008 ▶) *T*_min_ = 0.739, *T*_max_ = 0.867
6676 measured reflections
3720 independent reflections
2910 reflections with *I* > 2σ(*I*)
*R*_int_ = 0.034

#### Refinement

*R*[*F* ^2^ > 2σ(*F* ^2^)] = 0.038
*wR*(*F* ^2^) = 0.092
*S* = 1.03
3720 reflections
190 parameters
1 restraint
H-atom parameters constrained
Δρ_max_ = 0.64 e Å^−3^
Δρ_min_ = −0.79 e Å^−3^
Absolute structure: Flack & Bernardinelli (2000 ▶), 1096 Friedel pairs
Flack parameter: 0.039 (10)

### 

Data collection: *APEX2* (Bruker, 2009 ▶); cell refinement: *SAINT* (Bruker, 2009 ▶); data reduction: *SAINT*; program(s) used to solve structure: *SHELXS97* (Sheldrick, 2008 ▶); program(s) used to refine structure: *SHELXL97* (Sheldrick, 2008 ▶); molecular graphics: *ORTEP-3 for Windows* (Farrugia, 2012 ▶); software used to prepare material for publication: *PLATON* (Spek, 2009 ▶) and *publCIF* (Westrip, 2010 ▶).

## Supplementary Material

Crystal structure: contains datablock(s) I, global. DOI: 10.1107/S1600536813009070/cv5397sup1.cif

Structure factors: contains datablock(s) I. DOI: 10.1107/S1600536813009070/cv5397Isup2.hkl

Supplementary material file. DOI: 10.1107/S1600536813009070/cv5397Isup3.cml

Additional supplementary materials:  crystallographic information; 3D view; checkCIF report

## Figures and Tables

**Fig. 1.**
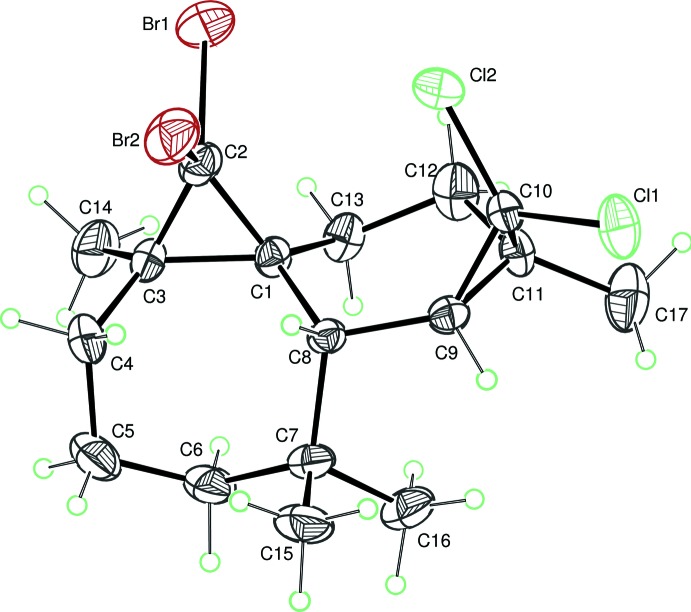
: Molecular structure of the title compound with the atom-labelling scheme. Displacement ellipsoids are drawn at the 50% probability. level. H atoms are represented as small spheres of arbitrary radii.

## supplementary crystallographic information

### Comment

As a continuation of our structural study of β-himachalene derivatives isolated from the essential oil of the Atlas cedar (Cedrus Atlantica) (Ourhriss *et al.*, 2013; Oukhrib *et al.*, 2013**a*,b*), we present here the crystal structure of the title compound, (1*S*,3*R*,8*R*,9S,11*R*)-2,2-dibromo-10,10-dichloro-3,7,7,11- tetramethyltetracyclo[6.5.0.0^1,3^.0^9,11^]tridecane, (I).

In (I) (Fig. 1), the molecule contains a fused six- and seven-membered rings, which are fused to two three-membered rings. The six-membered ring has a half chair conformation as indicated by the total puckering amplitude QT = 0.446 (5) Å and spherical polar angle θ = 140.8 (6)° and φ2 = 143 (1)°, whereas the seven-membered ring displays a boat conformation with QT = 1.122 (5) Å, θ2 = 87.4 (3)°, φ2 = -48.2 (3)° and φ3 = -118 (5)° (Cremer & Pople, 1975). The three-membered rings (C1/C2/C3) and (C9/C10/C11) are nearly perpendicular to the six and seven-membered rings (C1/C8–C13)and (C1/C3–C8), with a dihedral angles of 84.1 (4) and 80.6 (4), respectively. Owing to the presence of Br and Cl atoms, the absolute configuration could be fully confirmed from anomalous dispersion effects, by refining the Flack parameter as C1(S),C3(*R*),C8(*R*),C9(S), and C11(*R*). The crystal packing exhibits no short intermolecular contacts.

### Experimental

A solution containing 4 g (10 mmol) of (1*S*,3*R*,8*S*)-2,2-dibromo- 3,7,7,10- tetramethyltricyclo[6.4.0.01,3]dodec-9-ene and 1 ml (12.4 mmol) of CHCl_3_ in 40 ml of dichloromethane was added dropwise at 273 K over 30 min to 1 g (25 mmol) of pulverized sodium hydroxide and 40 mg of *N*–benzyltriethylam monium chloride placed in a 100 ml three–necked flask. After stirring at room temperature for 2 h, the mixture was filtered on celite and concentrated in vacuum. The residue obtained was chromatographed on silicagel column impregnated with silver nitrate (10%) with a mixture of hexane - ethyl acetate (96–4) used as eluent. The two diastereoisomers (1*S*,3*R*,8*S*,9S,11*R*)- 2,2-Dibromo-10,10-dichloro-3,7,7,11-tetramethyltetracyclo[6.5.0.0^1,3^.0 ^9,11^]tridecane(*X*) and its isomer (1*S*,3*R*,8*S*,9*R*,11*S*)-2,2-Dibromo-10,10- dichloro-3,7,7,11-tetramethyltetracyclo[6.5.0.0^1,3^.0^9,11^]tridecane(Y) were obtained by this procedure in a 80/20 ratio and a combined yield of 65% (3 g; 6.5 mmol). The title compound (isomer *X*) was recrystallized from hexane.

### Refinement

All H atoms were fixed geometrically and treated as riding with C—H = 0.96 Å (methyl),0.97 Å (methylene), 0.98 Å (methine) with *U*_iso_(H) = 1.2 *U*_eq_(methylene, methine) and *U*_iso_(H) = 1.5 *U*_eq_(methyl). The space group is non-centrosymmetric and the polar axis restraint is generated automatically by *SHELXL* program. The 1096 Friedel opposites reflections are not merged.

### Crystal data

**Table:**

C_17_H_24_Br_2_Cl_2_	*F*(000) = 460
*M**_r_* = 459.08	*D*_x_ = 1.677 Mg m^−^^3^
Monoclinic, *P*2_1_	Mo *K*α radiation, λ = 0.71073 Å
Hall symbol: P 2yb	Cell parameters from 3720 reflections
*a* = 9.0112 (6) Å	θ = 2.8–29.6°
*b* = 11.6772 (8) Å	µ = 4.75 mm^−^^1^
*c* = 9.0849 (6) Å	*T* = 296 K
β = 108.045 (5)°	Block, colourless
*V* = 908.94 (11) Å^3^	0.45 × 0.34 × 0.29 mm
*Z* = 2	

### Data collection

**Table:**

Bruker X8 APEX diffractometer	3720 independent reflections
Radiation source: fine-focus sealed tube	2910 reflections with *I* > 2σ(*I*)
Graphite monochromator	*R*_int_ = 0.034
φ and ω scans	θ_max_ = 29.6°, θ_min_ = 2.8°
Absorption correction: multi-scan (*SADABS*; Sheldrick, 2008)	*h* = −12→12
*T*_min_ = 0.739, *T*_max_ = 0.867	*k* = −16→15
6676 measured reflections	*l* = −12→12

### Refinement

**Table:**

Refinement on *F*^2^	Secondary atom site location: difference Fourier map
Least-squares matrix: full	Hydrogen site location: inferred from neighbouring sites
*R*[*F*^2^ > 2σ(*F*^2^)] = 0.038	H-atom parameters constrained
*wR*(*F*^2^) = 0.092	*w* = 1/[σ^2^(*F*_o_^2^) + (0.0333*P*)^2^] where *P* = (*F*_o_^2^ + 2*F*_c_^2^)/3
*S* = 1.03	(Δ/σ)_max_ = 0.001
3720 reflections	Δρ_max_ = 0.64 e Å^−^^3^
190 parameters	Δρ_min_ = −0.79 e Å^−^^3^
1 restraint	Absolute structure: Flack & Bernardinelli (2000), 1096 Friedel pairs
Primary atom site location: structure-invariant direct methods	Flack parameter: 0.039 (10)

### Special details

**Table:**

Geometry. All e.s.d.'s (except the e.s.d. in the dihedral angle between two l.s. planes) are estimated using the full covariance matrix. The cell e.s.d.'s are taken into account individually in the estimation of e.s.d.'s in distances, angles and torsion angles; correlations between e.s.d.'s in cell parameters are only used when they are defined by crystal symmetry. An approximate (isotropic) treatment of cell e.s.d.'s is used for estimating e.s.d.'s involving l.s. planes.
Refinement. Refinement of *F*^2^ against all reflections. The weighted *R*-factor *wR* and goodness of fit *S* are based on *F*^2^, conventional *R*-factors *R* are based on *F*, with *F* set to zero for negative *F*^2^. The threshold expression of *F*^2^ > σ(*F*^2^) is used only for calculating *R*-factors(gt) *etc*. and is not relevant to the choice of reflections for refinement. *R*-factors based on *F*^2^ are statistically about twice as large as those based on *F*, and *R*- factors based on all data will be even larger.

### Fractional atomic coordinates and isotropic or equivalent isotropic displacement parameters (Å^2^)

**Table:**

	*x*	*y*	*z*	*U*_iso_*/*U*_eq_	
C1	0.8734 (5)	0.4080 (3)	0.7265 (5)	0.0211 (8)	
C2	0.9989 (5)	0.3302 (4)	0.6990 (6)	0.0288 (10)	
C3	1.0234 (4)	0.3775 (4)	0.8568 (5)	0.0275 (9)	
C4	1.0081 (6)	0.2965 (4)	0.9812 (5)	0.0357 (11)	
H4A	1.1102	0.2657	1.0363	0.043*	
H4B	0.9413	0.2330	0.9329	0.043*	
C5	0.9408 (6)	0.3542 (5)	1.0962 (6)	0.0442 (13)	
H5A	1.0232	0.3954	1.1721	0.053*	
H5B	0.9014	0.2960	1.1507	0.053*	
C6	0.8095 (6)	0.4372 (5)	1.0187 (5)	0.0387 (12)	
H6A	0.8567	0.5027	0.9849	0.046*	
H6B	0.7670	0.4645	1.0981	0.046*	
C7	0.6702 (5)	0.3992 (4)	0.8798 (5)	0.0301 (9)	
C8	0.7181 (4)	0.3573 (3)	0.7345 (5)	0.0206 (8)	
H8	0.7319	0.2741	0.7439	0.025*	
C9	0.5812 (4)	0.3798 (4)	0.5883 (4)	0.0224 (8)	
H9	0.4807	0.3749	0.6086	0.027*	
C10	0.5657 (5)	0.3530 (4)	0.4239 (5)	0.0261 (9)	
C11	0.5826 (5)	0.4755 (4)	0.4748 (5)	0.0278 (9)	
C12	0.7378 (6)	0.5328 (4)	0.4939 (6)	0.0373 (11)	
H12A	0.7190	0.6125	0.4639	0.045*	
H12B	0.7856	0.4970	0.4234	0.045*	
C13	0.8528 (5)	0.5277 (3)	0.6570 (5)	0.0277 (9)	
H13A	0.9534	0.5556	0.6546	0.033*	
H13B	0.8168	0.5784	0.7235	0.033*	
C14	1.1485 (6)	0.4682 (5)	0.9207 (7)	0.0432 (13)	
H14A	1.1495	0.4896	1.0230	0.065*	
H14B	1.1262	0.5343	0.8546	0.065*	
H14C	1.2486	0.4377	0.9247	0.065*	
C15	0.5843 (6)	0.2992 (5)	0.9266 (6)	0.0453 (13)	
H15A	0.4981	0.2763	0.8394	0.068*	
H15B	0.5463	0.3228	1.0096	0.068*	
H15C	0.6547	0.2360	0.9601	0.068*	
C16	0.5593 (6)	0.5035 (5)	0.8432 (6)	0.0419 (12)	
H16A	0.6113	0.5673	0.8140	0.063*	
H16B	0.5305	0.5232	0.9333	0.063*	
H16C	0.4673	0.4849	0.7596	0.063*	
C17	0.4417 (7)	0.5546 (4)	0.4175 (7)	0.0479 (14)	
H17A	0.4693	0.6299	0.4593	0.072*	
H17B	0.3576	0.5255	0.4506	0.072*	
H17C	0.4095	0.5581	0.3065	0.072*	
Cl1	0.37818 (14)	0.30587 (10)	0.30591 (14)	0.0415 (3)	
Cl2	0.70977 (16)	0.28463 (12)	0.36234 (14)	0.0429 (3)	
Br1	1.11977 (6)	0.38295 (6)	0.57096 (7)	0.05382 (18)	
Br2	0.97449 (6)	0.16666 (4)	0.66883 (6)	0.04537 (16)	

### Atomic displacement parameters (Å^2^)

**Table:**

	*U*^11^	*U*^22^	*U*^33^	*U*^12^	*U*^13^	*U*^23^
C1	0.0167 (18)	0.022 (2)	0.023 (2)	−0.0038 (15)	0.0039 (16)	−0.0013 (15)
C2	0.019 (2)	0.034 (2)	0.033 (3)	−0.0019 (17)	0.0085 (19)	−0.0011 (18)
C3	0.0185 (18)	0.035 (2)	0.028 (2)	0.005 (2)	0.0054 (17)	0.004 (2)
C4	0.028 (2)	0.049 (3)	0.024 (2)	0.003 (2)	−0.0001 (19)	0.003 (2)
C5	0.044 (3)	0.066 (4)	0.021 (3)	−0.004 (3)	0.007 (2)	−0.002 (2)
C6	0.038 (3)	0.057 (3)	0.023 (3)	−0.001 (2)	0.013 (2)	−0.010 (2)
C7	0.029 (2)	0.038 (2)	0.028 (2)	0.0027 (19)	0.0153 (19)	−0.004 (2)
C8	0.0199 (18)	0.0217 (19)	0.022 (2)	0.0009 (15)	0.0096 (17)	0.0021 (15)
C9	0.0205 (18)	0.0241 (18)	0.024 (2)	−0.0013 (18)	0.0093 (16)	0.0001 (18)
C10	0.022 (2)	0.035 (2)	0.019 (2)	0.0020 (17)	0.0027 (17)	0.0016 (16)
C11	0.029 (2)	0.028 (2)	0.023 (2)	0.0031 (18)	0.0021 (19)	0.0076 (17)
C12	0.035 (3)	0.030 (2)	0.043 (3)	−0.006 (2)	0.007 (2)	0.011 (2)
C13	0.022 (2)	0.022 (2)	0.037 (3)	−0.0034 (17)	0.0067 (19)	0.0028 (18)
C14	0.023 (2)	0.054 (3)	0.047 (3)	−0.008 (2)	0.004 (2)	−0.005 (2)
C15	0.043 (3)	0.068 (3)	0.033 (3)	−0.005 (3)	0.024 (3)	0.004 (3)
C16	0.036 (3)	0.051 (3)	0.044 (3)	0.011 (2)	0.019 (3)	−0.008 (2)
C17	0.042 (3)	0.038 (3)	0.053 (3)	0.012 (2)	−0.001 (3)	0.003 (2)
Cl1	0.0330 (6)	0.0449 (7)	0.0341 (7)	−0.0066 (5)	−0.0077 (5)	−0.0011 (5)
Cl2	0.0438 (7)	0.0613 (8)	0.0262 (6)	0.0125 (6)	0.0144 (5)	−0.0040 (5)
Br1	0.0336 (3)	0.0840 (4)	0.0516 (3)	−0.0014 (3)	0.0244 (3)	0.0083 (3)
Br2	0.0452 (3)	0.0386 (2)	0.0525 (3)	0.0134 (3)	0.0156 (3)	−0.0033 (3)

### Geometric parameters (Å, º)

**Table:**

C1—C13	1.522 (6)	C9—C11	1.524 (6)
C1—C2	1.530 (6)	C9—H9	0.9800
C1—C3	1.537 (5)	C10—C11	1.496 (6)
C1—C8	1.542 (5)	C10—Cl2	1.756 (4)
C2—C3	1.488 (6)	C10—Cl1	1.785 (4)
C2—Br1	1.924 (4)	C11—C12	1.511 (6)
C2—Br2	1.932 (4)	C11—C17	1.525 (7)
C3—C4	1.513 (6)	C12—C13	1.524 (7)
C3—C14	1.524 (7)	C12—H12A	0.9700
C4—C5	1.519 (7)	C12—H12B	0.9700
C4—H4A	0.9700	C13—H13A	0.9700
C4—H4B	0.9700	C13—H13B	0.9700
C5—C6	1.523 (7)	C14—H14A	0.9600
C5—H5A	0.9700	C14—H14B	0.9600
C5—H5B	0.9700	C14—H14C	0.9600
C6—C7	1.544 (6)	C15—H15A	0.9600
C6—H6A	0.9700	C15—H15B	0.9600
C6—H6B	0.9700	C15—H15C	0.9600
C7—C15	1.531 (7)	C16—H16A	0.9600
C7—C16	1.545 (7)	C16—H16B	0.9600
C7—C8	1.587 (5)	C16—H16C	0.9600
C8—C9	1.529 (6)	C17—H17A	0.9600
C8—H8	0.9800	C17—H17B	0.9600
C9—C10	1.490 (6)	C17—H17C	0.9600
			
C13—C1—C2	118.6 (3)	C10—C9—H9	111.8
C13—C1—C3	119.8 (3)	C11—C9—H9	111.8
C2—C1—C3	58.1 (3)	C8—C9—H9	111.8
C13—C1—C8	112.1 (3)	C9—C10—C11	61.4 (3)
C2—C1—C8	120.5 (3)	C9—C10—Cl2	124.4 (3)
C3—C1—C8	118.0 (3)	C11—C10—Cl2	121.3 (3)
C3—C2—C1	61.2 (3)	C9—C10—Cl1	116.4 (3)
C3—C2—Br1	121.4 (3)	C11—C10—Cl1	117.8 (3)
C1—C2—Br1	119.6 (3)	Cl2—C10—Cl1	108.9 (2)
C3—C2—Br2	118.7 (3)	C10—C11—C12	117.4 (4)
C1—C2—Br2	123.5 (3)	C10—C11—C9	59.1 (3)
Br1—C2—Br2	106.9 (2)	C12—C11—C9	116.6 (4)
C2—C3—C4	117.9 (4)	C10—C11—C17	118.6 (4)
C2—C3—C14	119.7 (4)	C12—C11—C17	114.5 (4)
C4—C3—C14	112.2 (4)	C9—C11—C17	119.7 (4)
C2—C3—C1	60.7 (3)	C11—C12—C13	114.9 (4)
C4—C3—C1	117.3 (3)	C11—C12—H12A	108.5
C14—C3—C1	120.2 (4)	C13—C12—H12A	108.5
C3—C4—C5	112.6 (4)	C11—C12—H12B	108.5
C3—C4—H4A	109.1	C13—C12—H12B	108.5
C5—C4—H4A	109.1	H12A—C12—H12B	107.5
C3—C4—H4B	109.1	C1—C13—C12	113.5 (4)
C5—C4—H4B	109.1	C1—C13—H13A	108.9
H4A—C4—H4B	107.8	C12—C13—H13A	108.9
C4—C5—C6	112.5 (4)	C1—C13—H13B	108.9
C4—C5—H5A	109.1	C12—C13—H13B	108.9
C6—C5—H5A	109.1	H13A—C13—H13B	107.7
C4—C5—H5B	109.1	C3—C14—H14A	109.5
C6—C5—H5B	109.1	C3—C14—H14B	109.5
H5A—C5—H5B	107.8	H14A—C14—H14B	109.5
C5—C6—C7	120.9 (4)	C3—C14—H14C	109.5
C5—C6—H6A	107.1	H14A—C14—H14C	109.5
C7—C6—H6A	107.1	H14B—C14—H14C	109.5
C5—C6—H6B	107.1	C7—C15—H15A	109.5
C7—C6—H6B	107.1	C7—C15—H15B	109.5
H6A—C6—H6B	106.8	H15A—C15—H15B	109.5
C15—C7—C6	110.2 (4)	C7—C15—H15C	109.5
C15—C7—C16	108.2 (4)	H15A—C15—H15C	109.5
C6—C7—C16	104.9 (4)	H15B—C15—H15C	109.5
C15—C7—C8	106.7 (4)	C7—C16—H16A	109.5
C6—C7—C8	114.0 (3)	C7—C16—H16B	109.5
C16—C7—C8	112.8 (4)	H16A—C16—H16B	109.5
C9—C8—C1	113.2 (3)	C7—C16—H16C	109.5
C9—C8—C7	108.4 (3)	H16A—C16—H16C	109.5
C1—C8—C7	113.9 (3)	H16B—C16—H16C	109.5
C9—C8—H8	107.0	C11—C17—H17A	109.5
C1—C8—H8	107.0	C11—C17—H17B	109.5
C7—C8—H8	107.0	H17A—C17—H17B	109.5
C10—C9—C11	59.5 (3)	C11—C17—H17C	109.5
C10—C9—C8	129.4 (3)	H17A—C17—H17C	109.5
C11—C9—C8	122.7 (3)	H17B—C17—H17C	109.5
